# FLRTing Neurons in Cortical Migration During Cerebral Cortex Development

**DOI:** 10.3389/fcell.2020.578506

**Published:** 2020-09-17

**Authors:** Claudia Peregrina, Daniel del Toro

**Affiliations:** ^1^Department of Biological Sciences, Faculty of Medicine, Institute of Neurosciences, University of Barcelona, Barcelona, Spain; ^2^Institut d’Investigacions Biomèdiques August Pi i Sunyer (IDIBAPS), Barcelona, Spain; ^3^Centro de Investigación Biomédica en Red sobre Enfermedades Neurodegenerativas (CIBERNED), Madrid, Spain

**Keywords:** FLRT, Latrophilin, adhesion, repulsion, neuronal migration, Teneurin, Unc5

## Abstract

During development, two coordinated events shape the morphology of the mammalian cerebral cortex, leading to the cortex’s columnar and layered structure: the proliferation of neuronal progenitors and cortical migration. Pyramidal neurons originating from germinal zones migrate along radial glial fibers to their final position in the cortical plate by both radial migration and tangential dispersion. These processes rely on the delicate balance of intercellular adhesive and repulsive signaling that takes place between neurons interacting with different substrates and guidance cues. Here, we focus on the function of the cell adhesion molecules fibronectin leucine-rich repeat transmembrane proteins (FLRTs) in regulating both the radial migration of neurons, as well as their tangential spread, and the impact these processes have on cortex morphogenesis. In combining structural and functional analysis, recent studies have begun to reveal how FLRT-mediated responses are precisely tuned – from forming different protein complexes to modulate either cell adhesion or repulsion in neurons. These approaches provide a deeper understanding of the context-dependent interactions of FLRTs with multiple receptors involved in axon guidance and synapse formation that contribute to finely regulated neuronal migration.

## Introduction

The cerebral cortex is an evolutionary advanced structure with complex functionality that is organized in two main axes: radial (vertical) and tangential (horizontal) (Geschwind and Rakic, 2013). The radial axis results from the migratory direction of pyramidal neurons in relation to the pial surface. This neuronal migration follows an inside-out pattern and produces distinct cortical layers where specific neurons settle and differentiate based on their time of birth and migration dynamics. The tangential axis reflects the horizontal position of cortical neurons and segregates them into different functional areas that process specific sensory, motor and cognitive information (Herculano-Houzel et al., 2013). The horizontal coordinates of neurons is determined by both, the relative position of their progenitors in the germinal zone lining the lateral ventricles and their tangential dispersion that occurs while migrating radially. This process is limited in lissencephalic species (with smooth cortices such as rodents) (Noctor et al., 2001) but extensive in gyrencephalic species (with folded cortices such as ferrets and most primates) (Reillo et al., 2011).

Cortical neurons migrate through dense environments where they can display complex trajectories. During their journey from germinal zones to the cortical plate (CP), neurons integrate a combinatorial code of receptor and ligand interactions that are presented from three sources: neighboring neurons/radial glia fibers, extracellular matrix components (ECM) and diffusible cues. These interactions can trigger a variety of context-dependent cellular responses based on the arrangement of receptors and expression of signal transducers. Most extracellular cell guidance cues belong to the axon guidance-related protein families that control the wiring of the neural system by guiding axons to their appropriate target, and can act over short (cell-cell/substrate contact) or long range (diffusible cues), triggering either adhesion/attraction or repulsive functional responses (see Seiradake et al., 2016; Bellon and Mann, 2018; for recent reviews). Thus, several cues have a dual role in both axon guidance and cellular migration, where they display similar cooperation and crosstalk between different pathways and stabilization through redundant mechanisms, making both processes remarkably robust despite their enormous complexity.

In this review we will focus on the fibronectin leucine-rich repeat transmembrane proteins (FLRTs) that emerged as the first class of cell adhesion molecules (CAMs) with repulsive functions by heterophilic interactions (and thus also referred to as ReCAMs) (Seiradake et al., 2014). Recent data has revealed a remarkable variety of structural arrangements between FLRTs and their binding partners known to be involved in axon guidance and synapse formation (Seiradake et al., 2014; Jackson et al., 2015, 2016; Lu et al., 2015; del Toro et al., 2020). By manipulating FLRTs binding interactions, cell migration assays have shown that they act as a bimodal guidance system regulating both radial migration by short and long-range repulsive signals and tangential dispersion through adhesive interactions. FLRTs are therefore the first family of ReCAMs to be described as having dual-functionality during the migration of cortical neurons.

## FLRTs

**FIGURE 1:**
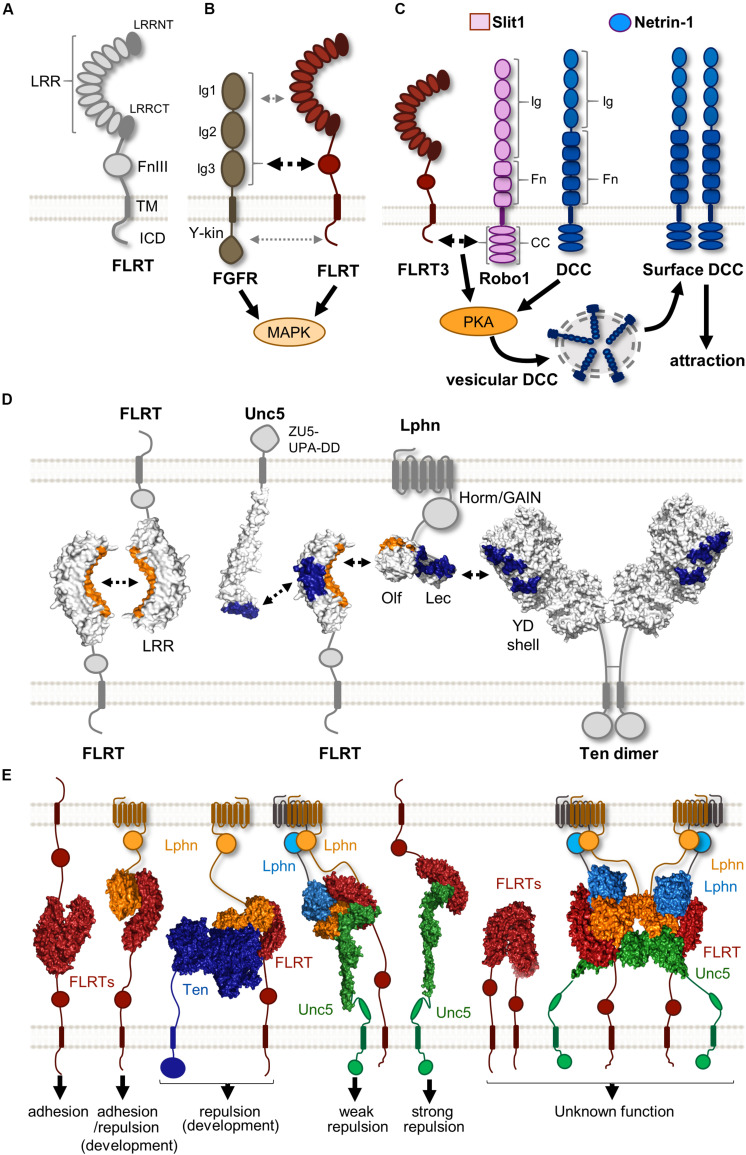
Protein complexes formed by FLRTs and their binding partners. **(A)** Scheme showing the structure of FLRTs proteins and their main domains. **(B)** FLRT interact with FGFR through its FNIII domain (black dashed arrow) regulating MAPK activity. Other interactions through FLRT LRR and ICD have been suggested (gray dashed arrow). **(C)** Scheme representing the mechanism by which responsiveness to Netrin-1 is achieved in rostral TCAs. In the presence of Slit1 and Netrin-1 FLRT-Robo interaction enhances PKA activity, which in turn increases surface levels of DCC, thereby enabling Netrin-1 attraction. **(D)** Scheme illustrating the known surfaces involved in FLRT LRR-dependent interactions. LRR domains participate in homophilic and heterophilic binding with different binding partners. Surfaces interacting with the concave site of the FLRT LRR domain are labeled in orange, whereas those binding the convex site are labeled in blue. **(E)** Overview of the variety of binary and ternary structure arrangements formed by FLRTs and its binding partners. These complexes cover a variety of responses ranging from adhesion to repulsion. The biological function of some of these structures remains largely unknown. These are selected examples of published protein structures (Seiradake et al., 2014; Jackson et al., 2015, 2016; del Toro et al., 2020).

The FLRT family protein comprises three members (FLRT1-3), all of which are type-I single-pass transmembrane receptor proteins involved in both repulsion and cellular adhesion depending on the cellular context and their binding partners. FLRT1 was the first member discovered around 20 years ago following an attempt to identify novel ECM components and interactors by screening a human skeletal muscle cDNA library (Lacy et al., 1999). The extracellular N-terminal region of all FLRTs contains 10 LRRs that are flanked by two highly conserved cysteine-rich regions and a fibronectin type III (FNIII) domain located adjacent to the membrane-spanning region by a linker containing a metalloprotease cleavage site (Yamagishi et al., 2011). FLRTs are glycosylated at 2 (FLRT1), 5 (FLRT2), or 4 (FLRT3) sites on their extracellular domains. A conserved sequence of 28 hydrophobic amino acids spans the cytoplasmic membrane (Figure 1A). The transmembrane helix connects to a relatively short non-homologous intracellular domain (ICD) that has been shown to interact with small Rho GTPases (Ogata et al., 2007) and modulate canonical fibroblast growth factor receptor (FGFR) signaling through the mitogen-activated protein kinase (MAPK) pathway (Böttcher et al., 2004; Wheldon et al., 2010).

## LRR-Independent Binding Partners

### FGFRs

Fibroblast growth factors (FGFs) and their receptors FGFRs (1-4) are known to regulate a plethora of developmental processes in the nervous system, including patterning, cell proliferation, survival, guidance, and synaptogenesis (Reuss and Von Bohlen Und Halbach, 2003; Salinas, 2005; Guillemot and Zimmer, 2011). FGFRs structure consists of an extracellular domain comprising of three immunoglobulin (Ig1, 2, and 3) domains, followed by a single transmembrane helix and an intracellular tyrosine kinase (Y-kin) domain (Dai et al., 2019; Figure 1B). At the cell surface, FGFRs have been shown to form large complexes involving various CAMs that modulate their signaling (Latko et al., 2019). FLRTs were first identified as modulators of FGF/FGFR signaling after the observation that both show similar expression patterns in many developing tissues of *Xenopus* (*African clawed frog*) (Böttcher et al., 2004) and mouse (Haines et al., 2006). Studies using fusion constructs have shown that all FLRT members interact with FGFR1, albeit with different affinities, by their FNIII domain (Böttcher et al., 2004; Haines et al., 2006), which is similar to the structural case between the Ig (2-3) motifs of FGFR1 and the FNIII domains of the neural cell adhesion molecule (NCAM) (Kiselyov et al., 2003). A later study using the yeast two-hybrid system also suggested that, in addition to the FNIII, both LRR and ICD domains contribute to the interaction between FLRT2 and FGFR2 (Wei et al., 2011; Figure 1B). This could explain the interaction between all FLRTs and a constitutively active form of FGFR1 where the entire extracellular region was replaced with the immunoglobulin FC domain (Haines et al., 2006), and the fact that the ICD of FLRT3 alone can promote FGF signaling (Böttcher et al., 2004). However, it is important to note that there is no structural data available for FLRT-FGFR interaction. All FLRTs have been shown to enhance FGF signaling by increasing the levels of phosphorylated ERK via MAPK activation (Böttcher et al., 2004; Wheldon et al., 2010). Interestingly, the ICD of FLRT1 contains three tyrosines that are targets for a FGFR1-dependent phosphorylation, which in turn potentiates the ability of FLRT1 to stimulate the ERK pathway. The functional consequence of such activation is the promotion of neurite outgrowth in mouse primary hippocampal neurons (Wheldon et al., 2010), which is consistent with previous studies showing that FLRT3 is upregulated after nerve injury and enhances neurite outgrowth (Robinson et al., 2004; Tsuji et al., 2004). Like FLRTs, other CAMs can stimulate neurite outgrowth such as neuroligins (NGLs) (Lin et al., 2003), and synaptic adhesion-like molecules (SALMs) (Wang et al., 2008).

### Robo

Robo (1-4) receptors are type-I single-pass transmembrane proteins. Their ectodomains are composed of five Ig and three FNIII domains (although Robo4 is smaller, containing only two of each domains), followed by a single transmembrane helix connected to a large ICD showing different conserved cytoplasmic (CC) motifs (Bisiak and McCarthy, 2019). Their ectodomain can trigger both repulsive and adhesive signaling depending on its binding partner. The classical Robo ligand, Slits (1-3), are best known for their function as chemorepellents during neuronal and axon guidance (Wu et al., 1999; Zhu et al., 1999; Ypsilanti et al., 2010) by binding to the Ig (1 and 2) domains of Robo receptors (Liu et al., 2004). This interaction is further stabilized by the addition of heparin sulfate (Fukuhara et al., 2008). However, their ectodomain can also mediate Robo homophilic interactions, mainly through the Ig domains, in a Slit-independent manner (Zakrys et al., 2014). These homophilic bindings trigger cell adhesion and stimulate neurite outgrowth (Hivert et al., 2002). Robo receptors participate in widely diverse functions during development due to their ability to interact with different co-receptors through forming both *cis* and/or *trans* extracellular interactions, as well as intracellular *cis*-interactions, thus creating crosstalk between several distinct signaling pathways (Bisiak and McCarthy, 2019).

One example of such interplay that has been studied in detail is the ability of Slit/Robo to regulate the Deleted in Colorectal Cancer (DCC) receptor signaling in a Netrin-1 dependent manner. In embryonic *Xenopus* spinal axons, activation of Robo1 by Slit leads to binding of its ICD to that of DCC, silencing Netrin-1 attraction (Stein and Tessier-Lavigne, 2001), which is consistent with the *in vivo* finding that postcrossing commissural axons acquire responsiveness to Slit, preventing their attraction to Netrin-1 (Reeber and Kaprielian, 2009). Interestingly, Slit/Robo can also enhance the attractive response to the guidance cue Netrin-1. The mammalian Robo3, which does not bind Slits because of mutations in the Ig1 domain, interacts with DCC via its ICD and is thereby phosphorylated when Netrin-1 binds DCC, thus potentiating Netrin attraction. Pontine neurons, lacking Robo3, phenocopy neurons deficient for DCC in their absence of attraction toward Netrin-1, suggesting that both receptors are required in mediating Netrin-1 dependent attraction of these neurons (Zelina et al., 2014). Further complexity in the regulation of the interplay between Slit/Robo and Netrin/DCC has been found during the development of thalamocortical axons (TCAs). Here, Slit1 enables Netrin-1 attraction in rostral TCAs, but exerts repulsion in intermediate TCAs, suggesting that Slit1 has a context-dependent role in TCA pathfinding and that combination of Slit1 and Netrin-1 differs from their individual effects (Bielle et al., 2011). Interestingly, both subsets of TCAs express similar levels of Robo (1 and 2), and the Netrin receptors, DCC and Unc5C, indicating that other co-receptors could participate in their molecular differences.

FLRT3 was found as a novel Robo1 interacting partner in a yeast two-hybrid screen using its ICD domain as a bait. There is a *cis*-interaction between FLRT3 and Robo1 ICDs and interestingly FLRT3 is expressed in TCAs in a rostral-to-caudal gradient (Leyva-Díaz et al., 2014). In the presence of Slit1 and Netrin-1, rostral TCAs expressing Robo1 and FLRT3 show upregulation of surface DCC through the activation of protein kinase A, which in turn induces Netrin-1 attraction (Figure 1C). Loss of FLRT3 or Robo1 in rostral TCAs prevents this effect, suggesting that both proteins are required to enable Netrin-1 sensitivity. Moreover, ectopic expression of FLRT3 in non-responsive intermediate TCAs that normally lack FLRT3, is sufficient to induce the attractive response observed in rostral TCAs in the presence of Slit1 and Netrin-1. This modulation of Netrin-1 responsiveness by FLRT3 is required for the proper navigation of TCAs to target different cortical areas, consistent with evidence of abnormal pathfinding of rostral TCAs in the absence of FLRT3 *in vivo* (Leyva-Díaz et al., 2014).

## Limited LRR-Binding Partners Still Form Multiple Protein-Protein Complexes

The LRR domain is the most studied ectodomain of FLRTs. It is present in a large number of proteins with diverse structure and function, being commonly found in proteins associated with the immune system and in neural development (Ng et al., 2011). The repeating nature of this domain, where LRR motifs array in tandem, results in a non-globular horseshoe-shape structure with two distinct surface areas: concave and convex, which correspond to the inner and outer circumference of the horseshoe, respectively (Kobe and Deisenhofer, 1994; Kajava, 1998). The increased surface area of this domain facilitates protein-protein interaction, and thus LRR- containing proteins have been implicated in intercellular communication and cell adhesion (Chen et al., 2006; Matsushima et al., 2007). In the nervous system, LRR-enriched proteins are highly expressed during development, showing diverse spatiotemporal expression patterns and roles in processes such as axon guidance, cellular migration and synapse formation (see de Wit et al., 2011; Schroeder and de Wit, 2018; for reviews). Here, we discuss the different LRR-dependent binding partners of FLRTs and the surprising variety of protein complexes that modulate several developmental processes.

### FLRT

The first LRR-dependent interaction responsible for mediating cell adhesion and sorting was found through direct FLRT-FLRT homophilic binding (Karaulanov et al., 2006). Structural data suggests that FLRTs dimerize via the concave surface of their LRR domain (Seiradake et al., 2014; Figure 1D), which is the common protein interaction surface on LRR domains (Kobe and Kajava, 2001). Supporting this notion, a single mutation in the concave surface reduces FLRT-FLRT interaction, based on multiangle light scattering (MALS) and cellular aggregation assays (Seiradake et al., 2014). We thereby named this mutant protein FLRT^FF^ (no FLRT-FLRT binding). Interestingly, there are different packing arrangements in FLRT-FLRT structures that all use the LRR concave surface, suggesting that FLRTs could multimerize rather than just dimerize. Indeed, the full ecto- and LRR domains of FLRT3 can oligomerize in a concentration-dependent manner (Seiradake et al., 2014), which could enhance the rather low-affinity nature of this interaction, as has been observed for other multimeric protein complexes (Hagner et al., 2018).

The homophilic *trans*-interaction between LRR domains could participate in different processes where FLRTs have been found to promote cell adhesion *in vivo* (Figure 1E). In the mouse, FLRTs are widely expressed in several tissues, except for FLRT1 that is restricted to the nervous system (Lacy et al., 1999). Embryos deficient for either FLRT2 or FLRT3 show lethality at earlier stages of development (around E10-E12) due to a wide range of malformations related to the formation and maintenance of tissue integrity, processes known to depend on cell adhesion mechanisms (Gumbiner, 1996). Absence of FLRT3 induces tissue disturbances that include headfold fusion and ventral closure defects leading to cardia bifida (Egea et al., 2008; Maretto et al., 2008), as well as disruptions to the basement membrane integrity of the anterior visceral endoderm (Egea et al., 2008), which is similar to those found in the basement membrane of the epicardium in FLRT2 mutant embryos (Müller et al., 2011). Interestingly, the phenotypes described in the absence of either FLRT2 or FLRT3 were found to be independent of FGF signaling (Egea et al., 2008; Maretto et al., 2008; Müller et al., 2011), suggesting that FLRTs mediate cell adhesion through other mechanisms or binding partners. Support for this idea comes from the finding that the FLRT LRR domain is dispensable for modulating FGF signaling (Böttcher et al., 2004), but essential for FLRT3-mediated cell sorting and aggregation (Karaulanov et al., 2006; Seiradake et al., 2014).

### Unc5

A further indication of the complexity of FLRT function comes from studies in *Xenopus* where FLRT3 was shown to interact with the small GTPase Rnd1, thus promoting cellular de-adhesion via downregulation of the CAM C-Cadherin, and thereby causing detachment of migrating equatorial cells (Ogata et al., 2007). A follow-up study to identify novel partners of the *Xenopus* FLRT3 ectodomain using a mouse embryonic cDNA library, found that the Netrin Uncoordinated-5 (Unc5) receptors, Unc5B and Unc5D, interact with high affinity to the LRR domain of FLRT3 (Karaulanov et al., 2009). Unc5B also interacts with Rnd1 and its expression enhances the de-adhesion effects of FLRT3 and Rnd1, suggesting that Unc5B modulates FLRT3 adhesive properties (Karaulanov et al., 2009). Like FLRTs, Unc5 receptors are type-I transmembrane protein, but their ectodomain structure differs radically, with two Ig (1 and 2) and two thrombospondin-like (TSP1 and 2) domains, followed by a transmembrane and cytoplasmic tail that contains ZU5, UPA and a death domain (DD) (Wang et al., 2009).

Uncoordinated-5 receptors are best known for their role in axon guidance triggering repulsion in response to Netrin-1, mainly through heterodimerization with DCC between their cytoplasmic domains (Hong et al., 1999; Finci et al., 2014), but also binding to Down Syndrome Cell Adhesion Molecule (DSCAM) through their ectodomains (Purohit et al., 2012). In addition to their roles in axon guidance, Unc5 receptors act as dependence receptors for Netrins, inducing apoptosis after cleavage of their intracellular DD domain in the absence of a ligand (Llambi et al., 2001), and also inhibit sprouting angiogenesis in a Netrin-1 dependent manner (Larrivée et al., 2007). The strong link between Unc5 receptor function and Netrin, contrast with the finding that some phenotypes observed in Unc5 null mouse models, such as trochlear nerve misprojetions in Unc5C- (Burgess et al., 2006) or increased vascular branching in the retina of Unc5B-deficient mouse (Koch et al., 2011), are not observed in embryos lacking Netrin-1. These results, together with the finding that Netrin is not present in several Unc5-expressing tissues in the mouse, such as the developing cortex, suggests the presence of other interactors. Supporting this notion is the finding that some Unc5 receptors bind to *Xenopus* FLRT3 promoting cellular de-adhesion (Karaulanov et al., 2009), raising the possibility that similar interactions could provide guidance to migrating cells and/or pathfinding axons in other organisms.

The work of Yamagishi and coworkers provided the first evidence that FLRT/Unc5 signaling regulates both neuronal migration and axon guidance by triggering repulsion in the mouse (Yamagishi et al., 2011). The full ectodomain of all FLRTs is shed from neurons by an unknown metalloprotease that cleaves near the plasma membrane, and binds to all Unc5 (A-D) receptors, albeit with different affinities. Thus, Unc5A/B prefer FLRT1, Unc5D prefers FLRT2 and Unc5B has higher affinity for FLRT3 (Yamagishi et al., 2011; Seiradake et al., 2014). Structural data showed that their binding interface involves the convex surface of the LRR domain of FLRTs and the most N-terminal domain of Unc5 receptors (Ig1 domain) (Figure 1D). This was further confirmed in surface plasmon resonance (SPR) and cell-based assays that showed the lack of binding between mutant proteins targeting these interactions domains, thus named as FLRT^UF^ and Unc5^UF^ (no Unc5-FLRT binding) (Seiradake et al., 2014). The FLRT/Unc5 interaction is likely to occur *in trans* (Figure 1E) because of the long stretched nature of the entire Unc5 ectodomain, the *in vivo* diffusion of the shed FLRT ectodomains (Yamagishi et al., 2011), and the frequent non-overlapping expression between FLRTs and Unc5 receptors in different tissues such as the cortex, hippocampus (Yamagishi et al., 2011) and retina during development (Visser et al., 2015).

Our studies focusing on FLRT/Unc5 signaling have shown that FLRTs trigger repulsion and growth cone collapse to Unc5-expressing neurons (Yamagishi et al., 2011). This response is induced by the ectodomains of FLRT and FLRT^FF^, but not FLRT^UF^, suggesting that it depends on FLRT-Unc5 interactions (Seiradake et al., 2014). A similar result to that has been observed in classical axon guidance protein families where both partners act as receptors, such as Eph/ephrin. There are, however, important differences between these two systems. Although one study suggested that Unc5C can repel a subpopulation of retinal neurons expressing FLRT2 (Visser et al., 2015), there is so far no evidence for FLRT/Unc5 bidirectional signaling, where Eph/ephrin complex signaling acts upon both Eph- and ephrin-expressing cells (Kania and Klein, 2016). Moreover, co-expression of Ephs and ephrins within the same cellular membrane can result in *cis*-interaction that reduces the number of receptors available for functional interaction, known as “*cis* inhibition” (Egea and Klein, 2007). This is in contrast to the FLRT/Unc5 system where rostral TCA that express both FLRT3 and Unc5B (Leyva-Díaz et al., 2014), do not show *cis* interaction, but rather parallel signaling where both FLRT3 and Unc5B at the cell surface can bind to exogenous FLRT3, and the adhesive FLRT interaction reduces the repulsive response triggered by FLRT-Unc5 interaction in a combinatorial way (Seiradake et al., 2014).

## Latrophilin

Latrophilins (Lphn1-3) were first identified as receptors for α-latrotoxin, a black widow spider toxin that results in activation of exocytosis mechanisms causing massive release of neurotransmitters from synaptic terminals (Krasnoperov et al., 1997; Lelianova et al., 1997). Lphn receptors are members of the G protein-coupled receptors (GPCRs) superfamily (Sugita et al., 1998), the largest and most diverse group of mammalian transmembrane proteins (Heifetz et al., 2015). They remained orphan receptors for several years, despite its expression being largely restricted to the brain for Lphn1 and 3 (Ichtchenko et al., 1999), and a proposed role in synaptic function (Südhof, 2001). All Lphns (1-3) show a similar structure comprising a large ectodomain (around 1000 amino acids) with lectin (Lec), olfactomedin (Olf), hormone receptor and GAIN domains, followed by the common feature of all GPCRs – the seven-pass transmembrane domain and the ICD (Lelianova et al., 1997; Sugita et al., 1998).

Fibronectin leucine-rich repeat transmembrane proteins were found to be endogenous ligands for Latrophilins by affinity chromatography coupled with mass spectrometry, using the ectodomain of Lphn3 fused to FC as a bait to identify binding partners from synaptosome extracts (O’Sullivan et al., 2012). All FLRTs were found to bind Lphn1 and 3 in *trans* through their ectodomains, and localized in hippocampal neurons to glutamatergic synapses. Disruption of FLRT3-Lphn3 binding by competition using their ectodomains or by knocking down either Lphn3 or FLRT3 reduced the density of glutamatergic synapses *in vitro* and *in vivo*, suggesting a role in synapse formation or maintenance as heterophilic CAMs (O’Sullivan et al., 2012). A follow-up study found that the Lphn3 Olf domain is required for this synapse-promoting function, as well as for FLRT3 binding (O’Sullivan et al., 2014). Structural data confirmed that the Lphn Olf domain interacts with the concave surface of the FLRT LRR domain (Lu et al., 2015; Ranaivoson et al., 2015), previously known to mediate homophilic FLRT binding, as observed by the lack of binding of FLRT^UF^ mutants to Lphns by SPR and cell binding assays (Jackson et al., 2015; Lu et al., 2015; Figure 1D). Stripe assays showed that Lphn3 promotes adhesion of HeLa cells expressing FLRT2, which supports the proposed role of Lphn3 in promoting synapse development (O’Sullivan et al., 2012). Surprisingly, the same experimental approach revealed that Lphn3 induces repulsion of cortical neurons that endogenously express FLRTs (Jackson et al., 2015). This repulsive effect depends on the binding of Lphn3 to FLRTs, since the non-FLRT-binding mutant, Lphn3^LT^, was unable to elicit repulsion. Therefore, this result could reflect the ability of FLRTs in *cis* recruitment of other receptors with repulsive activity, such as Robo1 (Leyva-Díaz et al., 2014) or Unc5 (Yamagishi et al., 2011) upon Latrophilin binding. Indeed, the Lphn3/FLRT3 structure showed that Lphn3 binds FLRT3 at a surface distinct from Unc5, and cell binding assays suggested that Latrophilin and Unc5 could simultaneously bind to FLRT3 (Lu et al., 2015).

Jackson and coworkers obtained the structure of the ternary complex Lphn/FLRT/Unc5 formed by their ectodomains (Lec-Olf/LRR/Ig1), revealing a stoichiometry of 1:1:2 (FLRT2:Unc5D:Lphn3) (Jackson et al., 2016; Figure 1E). Stripe assays showed that Lphn3 does not induce adhesion in HeLa cells expressing Unc5D and FLRT2, which contrasts to the strong adhesive response found in cells expressing FLRT2 alone or Unc5D with FLRT2^UF^ mutant that binds Lphn3 but not Unc5 (Jackson et al., 2016). These results suggest that Unc5D acts as a switch modulating the adhesive properties of FLRT2-Lphn3 interaction, resembling the finding that Unc5B regulates FLRT3 adhesive properties (Karaulanov et al., 2009). Interestingly, a complex comprised of FLRT2 LRR, Lphn3 Lec-Olf domains, and a larger ectodomain of Unc5D (Ig1Ig2TSP1), leads to the formation of an octamer through dimerization of the tetramer described above, and was the first example of a super-complex formed by three receptors involved in cell guidance (Jackson et al., 2016; Figure 1E). This structure showed a new binding interface between the Unc5D TSP1 and the convex side of FLR2 LRR domain, close to the binding site for Unc5D Ig1 domain. Although the function of the octamer is unclear, it could promote cell adhesion in different scenarios, such as maintenance of synaptic connections, similar to the finding that large protein complexes stabilize cellular contacts with the ECM (Wu, 2007).

Other known Latrophilin ligands involved in cellular adhesion include members of the Neurexin (Boucard et al., 2012) and Teneurin (Silva et al., 2011) protein family. Teneurins (Ten 1-4) are type-II single-pass transmembrane receptor proteins, strongly enriched in the nervous system where they play a role in synapse organization, neuronal migration and axon guidance (see Jackson et al., 2019 for a recent review). These diverse functions are thought to reflect their interactions with different binding partners. Indeed, Teneurins are characterized by a long modular C-terminal extracellular region that contains at least 16 domains. Some of the domains are involved in adhesive Teneurin homophilic interaction, such as the NHL domain (Berns et al., 2018), and others promote heterophilic binding, such as the *trans*-synaptic adhesion by engaging in *trans* interaction with Latrophilin (Silva et al., 2011; Boucard et al., 2014). This large ectodomain is followed by a transmembrane region and an N-terminal ICD. All Teneurins are localized at the cell surface and form *cis*-dimers through a covalent disulfide-link close to the plasma membrane (Feng et al., 2002).

Latrophilin was found to interact with FLRTs and Teneurins through two distinct domains: its Olf domain binds FLRT, while the Lec domain is mostly involved in the interaction with Teneurin (Boucard et al., 2014; O’Sullivan et al., 2014). This finding indicated that Latrophilins could interact simultaneously with FLRTs and Teneurins, similar to how FLRTs form a complex with Latrophilin and Unc5 receptors through distinct surfaces (Jackson et al., 2016). Consistent with this idea, it was found that Teneurin and FLRT located on the pre-synaptic site interact with the post-synaptic Latrophilin in *trans*. This coincident binding was necessary to induce synapse formation in hippocampal neurons *in vivo* (Sando et al., 2019). Recently, the structure of Latrophilin-Teneurin interaction has been solved, revealing that the Latrophilin Lec, and to a lesser extend Olf domain, bind across a spiraling beta-barrel domain of Teneurin, the YD shell (del Toro et al., 2020; Figure 1D). Structural superposition showed that the Latrophilin Olf domain can interact simultaneously with the Teneurin YD shell domain and the concave surface of the FLRT LRR domain (Figure 1E), thus suggesting the possibility of the formation of a Ten/Lphn/FLRT ternary complex. Evidence for physical interaction between these three proteins was found in a subset of embryonic cortical neurons expressing FLRT3 and Ten2, where both receptors show coincident binding to externally presented Latrophilin (del Toro et al., 2020). Strikingly, Latrophilin binding to Teneurins and FLRTs is repulsive for cortical neurons but not for their axons (del Toro et al., 2020), which contrasts with the adhesive/attractive nature of this interaction involved in synapse formation (Sando et al., 2019). Such a dual role in repulsive cell guidance and adhesive synaptogenesis has also been observed in the Eph/ephrin protein family (Kania and Klein, 2016; Henderson and Dalva, 2018), but the underlying mechanisms remain poorly understood.

## Cortical Migration

Neuronal migration is a tightly regulated and coordinated process that is essential for cortex development. During this phase, pyramidal neurons have to translate extracellular signals coming from substrates (neighboring neurons, ECM, radial glia fibers) and guidance cues into cytoskeletal arrangements and signal transduction modifications to follow their proper migratory route. This process is fundamental to establish the different cortical layers and appropriate cellular distribution, and thus alterations can lead to several types of cortical malformations including cortical heterotopias (subcortical and periventricular) and abnormal folding, such as lissencephaly, in humans (see Buchsbaum and Cappello, 2019; Subramanian et al., 2019 for recent reviews), that have been associated with several neuropsychiatric disorders such as schizophrenia and autism spectrum disorders (Fukuda and Yanagi, 2017; Guarnieri et al., 2018). Finally, recent studies in mouse and ferret models have demostrated the impact of neuronal migration on cortical folding. Genetic loss of doublecortin, a microtubule-stabilizing protein regulating radial migration, shows a lissencephalic phenotype in ferrets similar to human patients carrying mutations in this gene (Kou et al., 2015). Local knockdown of Cdk5 in upper cortical neurons, which modulates neuron migration and is mutated in some patients with lissencephaly, impairs radial migration and thereby affects the formation of folds in the ferret cortex (Shinmyo et al., 2017). In the mouse, genetic knockdown of genes modulating tangential dispersion of neurons such as EphAs/ephrinAs (Torii et al., 2009) and FLRTs (del Toro et al., 2017) results in cortical regions with neuronal segregation and heterogeneity inducing an uneven CP with alternating thicker and thinner areas, and in some cases can result in sulcus formation (del Toro et al., 2017).

## Radial Migration

**FIGURE 2:**
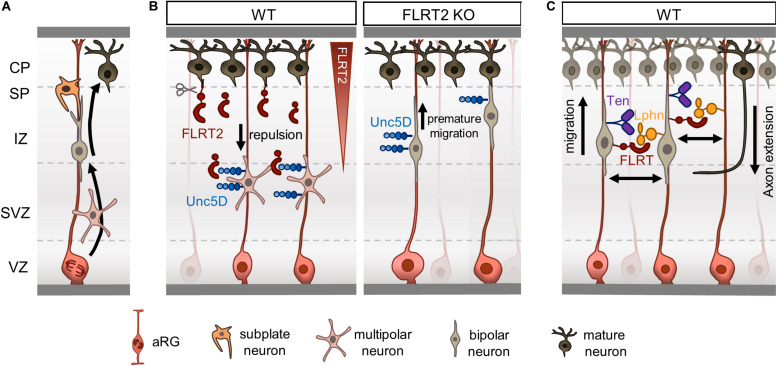
Fibronectin leucine-rich repeat transmembrane proteins (FLRTs) regulate radial migration by repulsion. **(A)** Overview of radial migration. Newborn neurons transition from multipolar to bipolar morphology during their migration to the CP. Subplate neurons facilitate the displacement of bipolar neurons to the CP by direct contacts. **(B)** FLRT2-Unc5D contacts regulate radial migration of neurons by a repulsive mechanism. FLRT2 knockout causes premature migration of Unc5D+ neurons toward the CP. **(C)** Schematic depicting a model where FLRT and Teneurin bind to Latrophilin in *trans* on opposing RG fibers or neurons.

Newborn pyramidal neurons are initially multipolar while moving radially from the SVZ and through the intermediate zone (IZ) to reach the CP. This initial phase of radial migration was initially described by Tabata and Nakajima (2003) and Tabata et al. (2009) after observing abundant migrating neurons populating the SVZ and lower portion of the IZ, with short processes mainly in the tangential axis without a defined polarity, and thus it was referred as multipolar migration. When moving, one of the thin processes of multipolar neurons elongates and gets thicker, becoming the so-called leading process that anticipates the direction of the movement, which can occur along both the radial and tangential axes and apparently seems independent of radial glial (RG) fibers. The elongation of the leading process is followed by nuclear translocation and retracement of the trail process, completing the locomotion movement (Marín et al., 2010). Multipolar neurons, characterized by random and low speed (1–3 μm/h) movements along the radial axis, transition to a bipolar morphology in the upper portion of IZ close to the subplate (SP), acquiring a fiber-guide locomotion mode characterized by fast migration speeds (9–12 μm/h) and strict radial orientation that causes their displacement to the CP (Tabata and Nakajima, 2003; Noctor et al., 2004). A recent study has shown that SP neurons facilitate such transition by inducing transient glutamatergic synaptic transmission to multipolar neurons that activates the calcium-dependent signaling required to modify their polarity and migration mode (Ohtaka-Maruyama et al., 2018; Figure 2A). Bipolar neurons entering the CP will migrate over earlier-born neurons residing in deeper layers to form superficial layers. The proteolytic processing of Reelin, a glycoprotein secreted mainly from Cajal–Retzius cells in the marginal zone (MZ), allows the formation of a gradient through the CP (Jossin et al., 2007). This gradient plays a critical role in the last steps of radial migration, directing the formation of cortical layers in an inside-out fashion, and acting as a stop signal to induce terminal translocation of migrating neurons beneath the MZ (see Hirota and Nakajima, 2017 for a detailed review).

Several systems such as cytoskeletal regulators, transcription factors and ECM molecules, have been identified to regulate radial migration and multipolar to bipolar transition. Due to diversity, and for further reading, we suggest reviews on this topic (Cooper, 2014; Ohtaka-Maruyama and Okado, 2015; Silva et al., 2019).

## FLRTs Regulate Radial Migration by Repulsion

The complexity of radial migration, where neurons transition through different phases and acquire a polarized structure with an established leading process that directs their movement, remarkably parallels the process of axon guidance, where immature neurons with short neurites designed to integrate both extrinsic cues and intrinsic mechanisms induce polarization (dendrites/axon) and axon path-finding. Indeed, several members of the four major classes of axon guidance cues (Eph/ephrins, Semaphorins, Netrins, and Slits) have been shown to participate in either cortical multipolar/bipolar migration or polarity by adhesion/attraction and repulsion mechanisms. Similarly, FLRTs modulate axon pathfinding and radial migration by triggering repulsion.

## FLRT-Unc5: Long-Range Repulsive Interaction

Both DCC and Unc5D are the only known Netrin receptors expressed by pyramidal neurons in the IZ that participate in their migration. Overexpression of DCC delays neuron migration in the IZ, an effect that can be rescued by increasing Unc5D levels, highlighting the need for a balance between levels of both receptors at the cell surface (Miyoshi and Fishell, 2012). Interestingly, Netrin-1 is not expressed in the developing cortex (Braisted et al., 2000), suggesting that other ligands could participate in its place. Unc5D is expressed in a subpopulation of pyramidal neurons that is marked by the expression of the non-coding *Svet1* RNA (Tarabykin et al., 2001). Indeed, *Svet1* RNA was found to be encoded by an intronic region of the unspliced RNA of Unc5D, and thereby a subset of multipolar neurons in the SVZ and IZ express both *Svet1* and Unc5D (Sasaki et al., 2008). Unc5D/*Svet1*-expressing neurons, born around E13.5 in the mouse, reside in the SVZ for an extended period before starting their migration. They begin entering the CP at E18.5 and finish their migration at P2 (Tarabykin et al., 2001). This migration to the CP is slower than other subpopulations born at the same time or even later. Unc5D/*Svet1*-neurons even reach the CP after Stab2-expressing neurons, which are born at around E14-E15, do not remain in the SVZ, and are present in the CP as early as E15.5 (Britanova et al., 2008).

Fibronectin leucine-rich repeat transmembrane protein 2, the main binding partner of Unc5D, is highly expressed in pyramidal neurons located in CP at E15.5. However, the ectodomain of FLRT2 is shed by an unknown metalloprotease and diffuses through the IZ to reach the SVZ where Unc5D/*Svet1* multipolar neurons linger (Yamagishi et al., 2011). Knockdown of FLRT2 accelerates the radial migration of Svet1-expressing neurons, while lack of Unc5D broadens the distribution of Tbr2-expressing cells, which also include Unc5D/Svet1-expressing neurons, toward the CP (Yamagishi et al., 2011; Figure 2B). These results are consistent with FLRT2 acting as repulsive cue for Unc5D-migrating neurons and suggests that both receptors participate in the delayed migration of a subset of pyramidal neurons. A follow-up study corroborated this finding by performing *in vivo* gain-of-function experiments using structure-based Unc5D proteins. Unc5D overexpression by *in utero*-electroporation (IUE) in pyramidal neurons born at E13.5 delayed their migration. This effect was partially rescued when expressing Unc5D^UF^ (Seiradake et al., 2014), supporting the notion that FLRTs participate in the radial migration of Unc5D/*Svet1* pyramidal neurons as repulsive cues.

The finding that FLRT2 acts as a diffusible guidance cue regulating radial migration is not without a precedent, and has been observed in other axon guidance protein families such as Semaphorins. Semaphorin 3A is a classical chemorepellent that regulates axon guidance by inducing growth cone collapse and turning by binding its co-receptors, Neuropilins, and Plexins (Manns et al., 2012; Koncina et al., 2013). During cortical development, Semaphorin 3A is highly expressed in the upper CP where it is secreted forming a gradient that attracts upper layer neurons, and thus promotes radial migration and proper orientation of the leading process of bipolar neurons toward the CP (Chen et al., 2008). Both co-receptors, Neuropilins and Plexins participate in radial migration. Acute knockdown of either Neuropilin-1 or PlexinA3, A4, and D1, which mediate Semaphorin 3A signaling, impairs radial migration (Chen et al., 2008). In addition, silencing of PlexinB2, but not B1, also impairs radial migration by altering RhoA activity that controls cytoskeleton dynamics in migrating neurons (Azzarelli et al., 2014).

## FLRT-Latrophilin-Teneurin: Contact-Repulsion Interaction

We recently showed that Latrophilins and Teneurins, known to promote synapse formation, are expressed in the cortex earlier in development, where they function in a complex with FLRTs to regulate radial migration by a contact-repulsion model (del Toro et al., 2020; Figure 2C). Like FLRTs, Teneurins are mainly expressed in pyramidal neurons in the mouse IZ and CP at E15.5, whereas Latrophilins show wider expression including the VZ, where apical RG cells show strong enrichment for Lphn1 and 2 (del Toro et al., 2020). A subset of cortical migrating neurons co-express FLRT3 and Ten2 that bind Latrophilins in *trans* on opposing RG cells or neurons, resembling the configuration proposed for their synaptogenic function (Sando et al., 2019). Stripe assays showed that Lphn1 induces repulsion of cortical neurons but not their axons. A double mutant Lphn1 that cannot bind FLRT and Teneurin, named Lphn1^TL–FL^ (no Ten-Lphn and FLRT-Lphn binding) did not elicit any response. The use of nanofibers mimicking RG fibers allowed the study of Lphn1 function in the context of neuron-RG fiber interaction. In these experiments, cortical neurons were found to migrate slower on nanofibers coated with Lphn1. This impairment was strongly reduced by using the double mutant Lphn1^TL–FL^. These results suggests that Lphn1 triggers repulsion through an additive or coincident interaction with Teneurins and FLRTs, which in turn affects neuronal migration (del Toro et al., 2020). Supporting the notion that Latrophilins act as a repulsive ligand during development, a recent study showed that Lphn2 repels Ten3-expressing hippocampal axons during target selection (Pederick et al., 2020). Lphn2 and Ten3 show complementary expression in the lateral hippocampal network, and knockdown of Lphn2 in the proximal subiculum results in an ectopic invasion of Ten3-expressing axons (Pederick et al., 2020).

Surprisingly, although FLRT3 and Ten2 show uniform distribution on the cell surface of cortical neurons, axons showed no response toward Lphn1 (del Toro et al., 2020). One possible explanation could be differences in the downstream signaling between the somatodendritic and axonal compartments. Similar results have been shown for Semaphorin 3A that facilitates the polarization of upper pyramidal neurons by attracting their apical dendrite toward the marginal zone (Polleux et al., 2000), while, through a repulsion response, it directs the growth of their axons toward the white matter (Polleux et al., 1998). Moreover, the highly polarized structure of migrating neurons could also contribute to the contrasting response between axons and dendrites. The leading process of migrating neurons preferentially interacts with the RG fibers (Elias et al., 2007). These increased contacts induces the polarized distribution of RhoA to the leading process, and Rac1 to the trail process that will become the axon (Xu et al., 2015).

*In vivo* overexpression or knockdown of Ten2 in cortical neurons delays their migration toward the CP. This effect is not observed when overexpressing the Ten2 mutant defective in Lphn binding, named Ten2^LT^ (no Lphn-Ten binding), suggesting that this response depends on Latrophilin interaction (del Toro et al., 2020). In support of this, disruption of endogenous interactions by competition using a secreted portion of the ectodomain of Lphn1 that binds FLRTs and Teneurins, but not its double mutant Lphn1^TL–FL^, also delays cortical migration (del Toro et al., 2020). The delayed migration observed when tampering with Ten2 levels on migrating neurons is reminiscent of other molecules regulating cortical migration, such as Rnd2 (Heng et al., 2008). In addition, FLRT loss- and gain-of-function experiments disturbs cortical migration (Seiradake et al., 2014). These studies suggest that excessive or reduced levels of proteins involved in adhesion or repulsion can be detrimental to cell migration. Indeed, the speed of cell migration can be reduced by modulating either adhesion or repulsion. Increased integrin-mediated cell-ECM adhesion (Haage et al., 2020), or reducing EphB-ephrinB contact repulsion reduces cell motility (Rohani et al., 2011). Conversely, increasing EphB-ephrinB repulsion induces cell detachment (Wen and Winklbauer, 2017), also affecting migration. Previous studies have identified different molecules promoting adhesion of migrating neurons to RG fibers such as connexin26/43 (Elias et al., 2007), focal adhesion kinase (Valiente et al., 2011), and N-cadherin (Shikanai et al., 2011). The molecules that mediate repulsion between neurons and RG fibers are not known, and thus Latrophilins are promising candidates to participate in this process.

Other interactions between Latrophilin, Teneurin, and FLRTs are conceivable. Migrating neurons expressing FLRTs and Teneurins could bind Latrophilins in *trans* on opposing RG fibers or neurons. Although it is possible that a subset of migrating neurons could express all three proteins, our results show that Lphn1 in *cis* does not abolish Ten2 or FLRT3 binding to exogenous Lphn1 (del Toro et al., 2020), which is similar to the finding that co-expression of FLRT3 and Unc5B do not induce *cis*-inhibition (Seiradake et al., 2014). Among all Teneurins, Ten4 showed mild expression in RG cells compared with Latrophilins (del Toro et al., 2020), which opens the possibility for an interaction in *trans* with Latrophilins on migrating neurons. Future studies using cell-specific manipulation of these proteins will help to elucidate the different context-dependent complexes that form as neurons migrate through their intricate environment.

## Tangential Distribution

**FIGURE 3:**
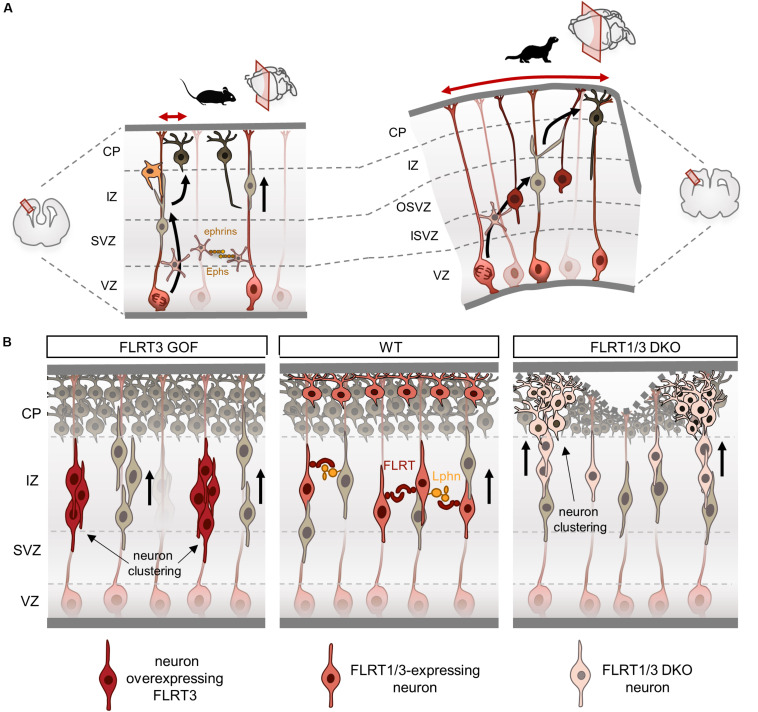
Fibronectin leucine-rich repeat transmembrane proteins (FLRTs) modulate tangential dispersion by adhesion. **(A)** Scheme illustrating tangential dispersion in the lissencephalic mouse and the gyrencephalic ferret during cortical development. In the mouse, pyramidal neurons mostly migrate radially along a single parent RG. Conversely, neurons in the ferret do not follow strict radial pathways and instead disperse in the lateral axis. **(B)** Current model of tangential dispersion modulated by FLRTs. In the wild-type (WT), FLRT1/3+ neurons show homogeneous distribution while migrating to the CP at E15.5. FLRT3 overexpression induces cell clustering in the IZ and delays migration. Loss of FLRT1/3 results in faster migration and clustering in the lower and upper CP. Loss of adhesion may alter tissue elasticity inducing a wavy upper surface of the CP, which can lead to sulcus formation.

There is a growing body of evidence showing substantial differences in cortical migration between lissencephalic and gyrencephalic species. Cell lineage analysis in clonal fashion of cortical progenitors have shown a striking diversity of migratory patterns during development. In rats, retroviral-labeling of progenitors at middle stages of development (E15-E16) produced neuronal clones that on average contained four cells spread along 250 μm (Luskin et al., 1993; Mione et al., 1994). In contrast, a similar approach in the ferret showed that neuronal clones labeled at middle-late neurogenesis (E33-35) contained large numbers of neurons with little tendency to cluster that can disperse several millimeters (from 1 mm up to 20 mm) in both the rostro-caudal and medio-lateral axes. These clonal-related neurons were found in different cortical regions such as the prefrontal, motor, somatosensory and visual areas, indicating that these cells are capable to disperse over large distances while acquiring different fates in functionally distinct cortical areas (Reid et al., 1997; Ware et al., 1999). In agreement with these findings, time-lapse experiments have shown that in the mouse, pyramidal neurons mostly migrate radially along a single parent RG fiber with little tangential spread (Noctor et al., 2001), whereas in folded brains like the ferret, migrating neurons do not follow strict radial pathways and instead disperse in the lateral axis leading to more convoluted migration routes concomitant with the start of cortical folding (Gertz and Kriegstein, 2015; Figure 3A).

Neurons use RG fibers as guides for migration and therefore changes in the radial fiber scaffold may contribute to neuronal migration differences between lissencephalic and gyrencephalic species. Indeed, gyrencephalic cortices show large numbers of basal RG cells, which account for the “fan-like” divergence of radial fibers, and therefore could facilitate lateral dispersion of migrating neurons (Reillo et al., 2011). Moreover, a recent study has shown that most of the bipolar neurons exhibit a branched leading process in the ferret, and to a lesser extent in the mouse. These branched processes are less parallel to radial fibers, do not affect radial migration, and importantly, seem to be involved in the remarkable lateral dispersion that occurs in folded cortices (Martínez-Martínez et al., 2019). Indeed, both the dynamic branching and filopodia formation that are observed in the leading process are similar features to those found in neurons exploring their environment during axon guidance (Dent et al., 2011). Together, these studies indicate that migrating neurons in folded cortices have increased cellular dynamics, exploratory behavior and lateral dispersion when compared to those in the rodent brain, but the nature of this mechanism remains largely unknown. Two axon guidance family proteins involved in cell adhesion and repulsion, Ephs/ephrins (Torii et al., 2009; Dimidschstein et al., 2013) and FLRTs (Seiradake et al., 2014; del Toro et al., 2017), have been shown to modulate tangential dispersion of migrating neurons in mouse cortices by adhesive mechanisms. Future work will be required to confirm whether changes in the adhesive properties of neurons allows them to acquire wide migratory profiles and tangential spread.

## FLRTs Modulate Tangential Dispersion by Adhesion

The pattern of expression of FLRT3/Unc5B in the mouse cortex at E15.5 is complementary to that of FLRT2/Unc5D, with FLRT3 expressed in migrating neurons in the IZ and Unc5B in the CP. The first insight into FLRT3 function in the cortex came from gain-of-function studies using structure-based FLRT3 proteins. *In vivo* overexpression of FLRT3 or FLRT3^UF^ delays neuron migration and alters their tangential distribution, forming a repeating pattern of aggregates in the IZ (Seiradake et al., 2014; Figure 3B). Conversely, overexpression of FLRT3^FF^ partially rescued the delayed migration induced by FLRT3 or FLRT3^UF^, and preserved the regular and homogeneous distribution of migrating neurons in the tangential axis. These results indicate that binding of FLRT3 to other ligands through the concave site of its LRR domain directs tangential distribution. One possible interaction could be FLRT3-FLRT3 homophilic binding, which induces *in vitro* cell aggregation and sorting (Karaulanov et al., 2006; Seiradake et al., 2014). The increased adhesion between overexpressing neurons could cause those cells to aggregate, and thereby result in delayed migration and segregation from surrounding cells. Indeed, ephrinB1 overexpression, which can induce cell homoadhesion (Batlle and Wilkinson, 2012), reduces the horizontal dispersion of multipolar neurons (Dimidschstein et al., 2013). Likewise, EphA/ephrinA gain-of-function experiments show reduced lateral dispersion of multipolar neurons and increased aggregation that alters the proper mixing of pyramidal neurons found in the cortical columns (Torii et al., 2009).

Several studies in CAMs that mediate homophilic binding, support a role for differential adhesion in cell segregation, such as cadherins (Nose et al., 1988; Steinberg and Takeichi, 1994). Interestingly, in *Xenopus*, FLRT3 has been shown to regulate C-cadherin surface expression by binding Rnd proteins through its ICD (Ogata et al., 2007; Karaulanov et al., 2009). A later study found that FLRT3 forms a complex with Paraxial protocadherin and C-cadherin regulating cell adhesion and sorting (Chen et al., 2009). There are therefore other possibilities, such as FLRT3 regulating surface expression and function of N-cadherin that participates in cortical migration (Kawauchi et al., 2010), or Rnd activity, which is known to regulate cell adhesion to the ECM (Guasch et al., 1998; Nobes et al., 1998), as well as radial migration in the developing cortex (Heng et al., 2008; Pacary et al., 2011; Azzarelli et al., 2014). N-cadherin controls cell migration either by regulating actin-myosin contractile forces (Shih and Yamada, 2012) or modulating FGFR-dependent signaling (Nguyen et al., 2019). During cortical migration, N-cadherin interacts in *cis* with FGFR1-3 at the cell surface of multipolar neurons, preventing their degradation. Thus, FGFRs accumulate and enhance their signal to the ERK pathway that is required for proper multipolar neuron migration and transition to bipolar cells (Kon et al., 2019). Given that N-cadherin has been shown to interact with the first two Ig domains of FGFR1 (Suyama et al., 2002), it is possible that both FLRT3 and N-cadherin compete for FGFR binding. Thus, the delayed neuron migration observed after FLRT3 overexpression could be due, in part, to altered regulation of N-cadherin-FGFR-dependent signaling. Finally, the recent finding that Latrophilins are expressed in the cortex in both neurons and RG fibers (del Toro et al., 2020), suggests the possible involvement of FLRT3-Lphn interaction directing the lateral distribution of migrating neurons.

Altered tangential distribution is also observed when FLRT3 expression is ablated in migrating neurons (Seiradake et al., 2014). Neurons lacking FLRT3 show abnormal cell clustering in the lateral portion of the cortex within the lower CP. Interestingly, FLRT3-expressing neurons also express FLRT1, which shares similar features in terms of homophilic adhesion and heterophilic binding to Unc5 and Latrophilins (Yamagishi et al., 2011; Seiradake et al., 2014; del Toro et al., 2020). Double deletion of FLRT1 and FLRT3 enhances the clustering effect observed in FLRT3 mutants, extending into medial and caudal regions of the cortex following a repeated pattern (del Toro et al., 2017; Figure 3B). Neurons deficient for FLRT1 and FLRT3 migrate faster and also segregate into clusters in the lower CP that extend into the upper CP as they migrate. This heterogeneity results in a wavy surface of the upper CP that can lead to sulcus formation (del Toro et al., 2017). FLRT-expressing cortical neurons aggregate *in vitro*, but not those deficient for FLRT1 and FLRT3 (del Toro et al., 2017), indicating that the effects of FLRT1/3 ablation *in vivo* are likely non-cell autonomous and may be the result of repulsive interactions with surrounding cells. A similar scenario is seen in the EphB2-ephrinB1 dependent repulsion, where EphB2 cells show increase migration speed during heterotypic repulsion and segregate from those expressing ephrinB1 (Taylor et al., 2017).

The tangential clustering and uneven CP found in FLRT1/3 ablated cortices resembles the phenotype seen in the ephrinA2/A3/A5 mutants, where neuronal segregation in the tangential axis leads to a CP with alternating thicker and thinner areas (Torii et al., 2009). In both mouse models, cell proliferation is not affected and therefore suggests that mechanical factors could influence the morphology of the CP. Supporting this notion, a recent study has shown that manipulation of the ECM can induce folding of the CP by modifying ECM stiffness (Long et al., 2018). Sulcus regions tend to have lower stiffness compared to gyrus areas, suggesting that modulation of local tissue stiffness could participate in the induction of folds in the cortex (Long et al., 2018). Therefore, the segregation and reduced intercellular adhesion of FLRT1/3 ablated neurons could contribute to forming a CP with non-homogeneous tissue elasticity, which in turn favors sulcus formation. Indeed, both FLRT1 and FLRT3 are less abundant in the cortical area that will form the lateral sulcus compared with the splenial gyrus in the ferret (del Toro et al., 2017). The finding that genetic mouse models that alter the morphology of the CP through altered tangential dispersion target at least two genes, such as FLRT1/3 (del Toro et al., 2017) or ephrinA2/A3/A5 (Torii et al., 2009) mutants, suggests the presence of redundant mechanisms that regulate the delicate balance of adhesion/repulsion required for cell migration (Solecki, 2012; Cooper, 2013).

## Concluding Remarks

During the last decade, FLRTs have been found to interact with different ligands governing a wide-repertoire of biological functions such as axon guidance, cell migration and synapse formation. Structural data has revealed a rich variety of protein complexes formed by FLRTs and its binding partners, which modulate the finely tune adhesive and repulsive cellular responses required for nervous system development. The combination of structural biology with cellular assays and the use of conditional knockout mouse models has shed light on how FLRT proteins are mechanistically involved in such a wide range of developmental processes.

The functions of FLRT proteins are best understood in the context of cortical migration during brain development. FLRTs participate in radial migration through at least two distinct mechanisms. FLRT2 acts as a long-range cue, where its ectodomain is shed from the CP triggering repulsion of Unc5-expressing neurons in the SVZ (Yamagishi et al., 2011). FLRT3 and Ten2 act in close contact with another neuron or RG fiber expressing Latrophilin, regulating neural migration by repulsion (del Toro et al., 2020). Conversely, FLRT proteins modulate tangential dispersion by adhesion, where homophilic and perhaps heterophilic interactions with Latrophilin are involved (Seiradake et al., 2014).

These studies elegantly illustrate the full strength of structure-function studies, and how structure-based analysis of mutant proteins can overcome that challenging nature of dissecting the *in vivo* functionality of specific protein complexes. Similar approaches can be used to investigate further interactions, such as the supercomplexes formed by Unc5, FLRT and Latrophilins (Jackson et al., 2016) and the possible role of Unc5 receptors in the context of FLRT-Latrophilin-Teneurin complex.

## Author Contributions

Both authors wrote and edited the manuscript.

## Conflict of Interest

The authors declare that the research was conducted in the absence of any commercial or financial relationships that could be construed as a potential conflict of interest.
